# Multiple Penetrating Eye Wounds Due to Suspected Self-Injury

**DOI:** 10.4274/tjo.galenos.2020.69822

**Published:** 2021-02-25

**Authors:** José Dalma-Weiszhausz, José Arturo Oyervides Alvarado, Ana Maria Suarez Licona, Miriam Tatiana Serment Azuara, Alejandro Dalma Kende

**Affiliations:** 1 Asociación para Evitar la Ceguera en México, Ciudad de México, México; 2 Instituto Nacional de Psiquiatría Ramón de la Fuente, Ciudad de México, México; 3 Dalma & Asociados, SC. Cuidad de México, México

**Keywords:** Self-injurious behavior, self-mutilation, substance abuse, psychotic symptoms, self-injury, oedipism

## Abstract

We present a case of a 29-year-old male night watchman complaining of sudden decreased vision, redness, and tearing of the left eye. On anamnesis, trauma was denied and personal past history was relevant for right eye enucleation due to an “eye injury” 8 years prior. At presentation, his visual acuity was 20/200 and intraocular pressure (IOP) was 10 mmHg. Slit lamp examination revealed a 1-mm inferonasal corneal wound and a localized lens opacity associated with extrusion and posterior extension of cortical material in the same quadrant. Echography confirmed posterior lens capsular bag puncture with hyperechogenic material in the anterior vitreous. Intraocular foreign body was ruled out. Topical anti-inflammatory and cycloplegic treatment was initiated with partial visual recovery, IOP rise, moderate anterior chamber inflammatory reaction, and an emergent posterior subcapsular cataract. A pars plana vitrectomy and lensectomy were performed. After surgery and recovery, best-corrected visual acuity with contact lens was 20/15. The patient was followed for 6 years, during which he returned 6 more times with a variety of new findings, such as new corneal leukoma, leaking corneal wounds, hypotony, choroidal folds, and choroidal detachments, each time with full visual acuity recovery. Some cases of ocular injury and self-mutilation have been described in the context of various psychiatric disorders. Self-inflicted injuries were suspected due to substance abuse, although the patient denied doing so. Referral to a psychiatrist was insisted on several occasions without success. However, potentially life-threatening complications may arise; therefore, psychiatric referral is imperative.

## Introduction

There are few reports in the ophthalmic literature regarding eye wounds as manifestation of self-injury. The incidence of self-enucleation, usually referred as oedipism^1,2^, is estimated at 1 in 30 million per year.^1,2^ Ocular self-injury has been related to religious beliefs and guilt (as mentioned in the gospel of Matthew 5:29).^2,3^ Ocular self-injury may also occur during a psychotic episode, usually due to schizophrenia, and has also been reported in the context of substance abuse, mood disorders, and other organic diseases.^1,2,3^

## Case Report

**Figure 1:**
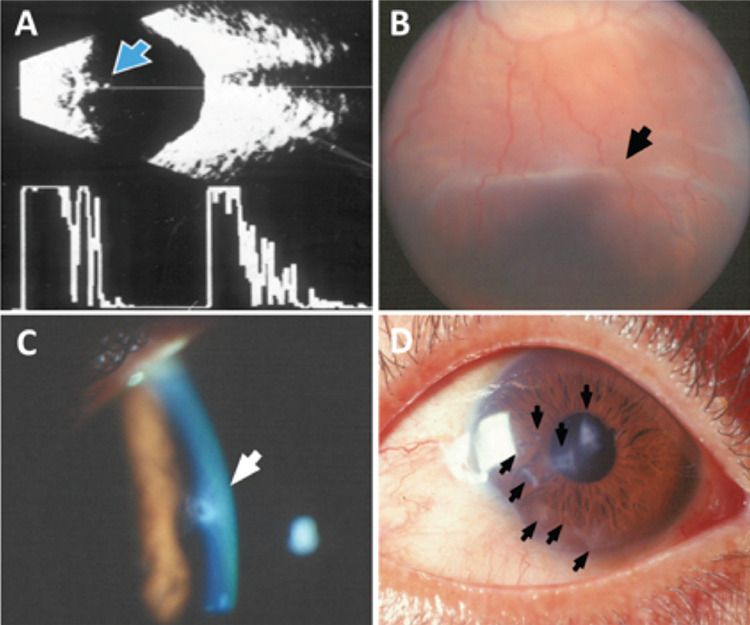
A) Echography of the left eye showing hyperechogenic material on the anterior vitreous (blue arrow). B) Fundus photography showing inferior choroidal fold due hypotony (black arrow). C, D) Anterior segment photography showing central corneal wound with positive Seidel test (white arrow) and multiple corneal leukomas from previous wounds (black arrows)

A 29-year-old Hispanic man employed as a night watchman presented for the first time complaining of acute red, tearing left eye with decreased vision. Recent eye trauma was denied. Personal past ophthalmologic history was relevant for right eye enucleation 8 years prior due to an unspecified “eye injury” with no available medical records. Past medical history was non-relevant. On examination, visual acuity was 20/200. A 1-mm self-sealing inferonasal corneal wound was noted with a small, localized lens opacity and posterior extrusion of cortical material in the same quadrant. Intraocular pressure (IOP) was 10 mmHg. Funduscopic examination was normal with no intraocular foreign body. Echography confirmed a posterior lens capsule rupture with cortical material in the anterior vitreous and no foreign body (Figure 1a). Topical anti-inflammatory and cycloplegic medication was initiated. After 1 week, vision improved to 20/50. A posterior subcapsular cataract developed with moderate anterior chamber inflammatory reaction and IOP of 25 mmHg. Lens-induced glaucoma was diagnosed and pars plana vitrectomy with lensectomy was performed without complications. No intraocular lens was implanted due to the ambiguity in the pathogenesis of the injury. Visual acuity of 20/25 with contact lens aphakic correction was achieved 2 weeks after surgery.

Two years passed uneventfully, until the patient presented with a small intrastromal perilimbal culture-negative corneal abscess. It was thought to be contact-lens related and treated with topical antibiotics. After recovery, visual acuity was 20/15 with a new contact lens. He was seen again 4 months later complaining of sudden decrease in visual acuity. On examination, visual acuity was 20/200 and IOP was low. Corneal leukomas from previous injuries were observed. No open wound was found. Funduscopic examination revealed an inferior choroidal fold (Figure 1b), 360º choroidal detachment and mild disc edema. He was treated with systemic prednisone, topical steroid and cycloplegic, with full recovery of vision. Three new, small, inferior, partial-thickness corneal leukomas were found in a routine 1-year follow-up visit. He firmly denied any traumatic events.

After 12 months, the patient was seen again reporting sudden vision loss. A Seidel-positive central corneal wound (Figure 1c) with a mild anterior chamber inflammatory reaction and a small iris wound in the superotemporal quadrant were documented. No intraocular foreign body was found. Antibiotics, anti-inflammatory medication, and a soft contact lens were prescribed. The episode resolved with vision returning to 20/15. At this point, repeated self-induced injuries were suspected. A psychiatric evaluation was insisted on but never done.

**Figure 2:**
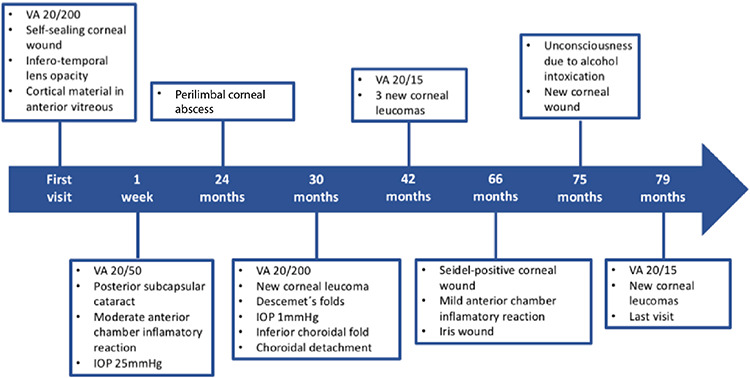
Time line of the patient’s follow-up, including all visits and most important findings VA: Visual acuity, IOP: Intraocular pressure

Nine months later, the patient was found unconscious due to acute alcohol intoxication. When he regained consciousness, he immediately started complaining of decreased vision. A complete examination was performed and a new corneal wound was found. In this visit, the patient admitted to frequent alcohol abuse. In the last visit, 8 corneal wounds were documented (Figure 1d). The patient never acknowledged the probable self-inflicted nature of the wounds. Psychiatric consultation was insisted upon on several occasions, but was never carried out. He was lost to follow-up after 6 years as shown in the timeline (Figure 2).

## Discussion

Unexplained eye injuries, especially when repeated, should alert us to the possibility of self-injury and/or abuse. To date, most cases of ocular injury and self-mutilation have been described in the context of psychiatric disorders. This behavior has been associated with several psychiatric conditions including psychotic symptoms (related to schizophrenia), obsessive-compulsive spectrum disorders, corporal dysmorphic disorders, affective disorders (bipolar and psychotic depression), and post-traumatic stress disorders.^5^

The prevalence of psychotic symptoms in the general population is variable, being higher in males.^2^ Mean age of presentation is 25 (±10) years.^2^ Substance-induced psychotic symptoms are most commonly associated with phencyclidine, lysergic acid diethylamide (LSD), cocaine, cannabis, and amphetamine use, but it has also been associated with alcohol abuse.^1,3^ Even though our patient could have had a primary psychotic disorder, the most likely trigger of his behavior was alcohol abuse.

Substance-induced psychotic symptoms could be associated with intoxication and deprivation phases. They tend to be transient with predominance of auditory and visual hallucinations.^4^ The psychotic symptoms related to alcohol use are more common in deprivation phase as a part of delirium tremens syndrome.^5^

One major cause of psychotic symptoms is schizophrenia, which has been associated with ocular self-inflicted injuries. Schizophrenia is characterized by insidious and gradual presentation of positive and negative symptoms, disorganized conduct, lack of judgment, and decline in cognitive function. Positive symptoms generally involve delusions of self-harm, mystic-magic themes, and hallucinations that could be punitive, aggressive, or disorganized. Sometimes, self-injury or self-mutilation is the result of these hallucinations. Taking into consideration the age and gender of the patient and the characteristics of the injuries, it is highly possible that he could have been going through a psychotic episodes, perhaps associated with substance abuse. It is highly likely that his other eye was lost in the same way.

Self-inflicted ocular injuries are associated with serious complications such as retinal detachment, endophthalmitis, damage to the optic chiasm, sympathetic ophthalmia, meningitis, subarachnoid hemorrhage ^3^, cerebrospinal fluid leakage, and loss of pituitary function.^5^ The ophthalmologist may be in a unique position to identify these patients and help them receive the multidisciplinary treatment they need to prevent vision loss, self-harm, and death. Pharmacological medications for psychotic symptoms, like risperidone, olanzapine, or aripiprazole are preferred. In some cases, when the self-injury and/or self-mutilations threaten function or life, hospitalization is indicated to ensure the patient’s and their relatives’ safety.^2,4^
